# Not Seeing Double: Discordance in Disease, Function, and Their Longitudinal Associations in Monozygotic Twins

**DOI:** 10.1093/geroni/igab046.2787

**Published:** 2021-12-17

**Authors:** Elizabeth Teas, Olivia Robertson, Kristine Marceau, Elliot Friedman

**Affiliations:** 1 Purdue University, West Lafayette, Indiana, United States; 2 Purdue University, Purdue University, Indiana, United States

## Abstract

Prior research on the causality and directionality between disease and functional limitations is ambiguous. The current study used longitudinal monozygotic twin data to test both directions linking disease burden and functional limitations in middle-aged and older adults, controlling for genetic and familial factors. We also examined potential moderation by psychological well-being. The Twins sub-sample from the first two waves of the longitudinal Midlife in the United States (MIDUS) study was used (Wave 1: 1995-1996, Wave 2: 2004-2006). Only monozygotic twins (N = 713) were included in analyses. In separate multi-level models, we examined disease burden at MIDUS 2 predicted by functional limitations at MIDUS 1 and MIDUS 2 functional limitations predicted by disease burden at MIDUS 1. Disease burden and functional limitations at MIDUS 2 varied substantially within families. There was no within-family association of earlier functional limitations with change in later disease burden (b = .40, p = .39), but there was a within-family association such that the twin with higher baseline disease burden had a greater increase in functional limitations than his/her co-twin (b = .06, p = .02). Well-being was not a moderator in either model. We found support for a potentially causal association between earlier disease burden and later increases in functional limitations, consistent with the Disablement Process Model. Sensitivity analyses confirm the detected within-family effect. Possible mechanisms linking disease burden and functional limitations are discussed as potential targets for future research.

